# Restoration of thymic T-cell development by bone marrow transplantation in mouse radiation lymphomagenesis

**DOI:** 10.1093/jrr/rrae045

**Published:** 2024-06-19

**Authors:** Tsuguhide Takeshima, Sumitaka Hasegawa

**Affiliations:** Department of Charged Particle Therapy Research, National Institutes for Quantum Science and Technology, Chiba 263-8555, Japan; Department of Charged Particle Therapy Research, National Institutes for Quantum Science and Technology, Chiba 263-8555, Japan

**Keywords:** radiation carcinogenesis, thymic lymphoma, Bone marrow transplantation, T-cell development, cancer prevention

## Abstract

Fractionated total body irradiation (TBI) with X-rays induces thymic lymphoma/leukemia (TL) in C57BL/6 mice. Radiation-induced mouse TL (RITL) can be prevented by bone marrow transplantation (BMT) of unirradiated BM cells. However, the mechanisms underlying the prevention of RITL with BMT remain unclear. Here, we show that BMT restores thymic T-cell differentiation in mice subjected to TBI. TBI (four times of 1.8 Gy X-rays weekly) was conducted with C57BL/6 mice. BMT was performed immediately after the last irradiation of TBI in mice by transplantation of BM cells isolated from enhanced green fluorescence protein (eGFP) transgenic mice. Thymic cell numbers were drastically decreased in TBI and TBI + BMT mice compared to those in non-irradiated mice. Flow cytometry showed a dramatic decrease in double negative (DN, CD4^−^CD8^−^) thymocytes, especially DN2 (CD25^+^CD44^+^) and DN3 (CD25^+^CD44^−^) subpopulations, in the TBI mice on Day 10 after the last irradiation. In contrast, the DN2 and DN3 populations were recovered in TBI + BMT mice. Interestingly, these restored DN2 and DN3 cells mainly differentiated from eGFP-negative recipient cells but not from eGFP-positive donor cells, suggesting that transplanted BM cells may interact with recipient cells to restore thymic T-cell development in the RITL model. Taken together, our findings highlight the significance of restoring thymic T-cell differentiation by BMT in RITL prevention.

## INTRODUCTION

Radiation-induced thymic lymphoma/leukemia (RITL) is a mouse model that has been intensively studied to elucidate the mechanism of radiation carcinogenesis since its first report about 70 years ago [1]. In the RITL model, fractionated total body irradiation (TBI) with X-rays induces RITL in over 90% of C57BL/6 mice after a latency period of several hundred days from the first irradiation [2].

Several findings have been reported regarding the pathogenesis of RITL. TBI induces thymic atrophy and aberrant thymic development occurs after TBI in an RITL mouse model [3]. Bone marrow transplantation (BMT) can prevent RITL, but this critically depends on the timing of BMT administration. BMT within 1 day after completion of fractionated TBI could reduce RITL, but delayed BMT at 10 or 30 days after TBI could not [4]. Shielding the BM from radiation suppresses RITL development, even when the thymus is irradiated [5]. RITL development could be prevented by thymectomy in mice receiving fractionated TBI and subcutaneous grafting of unirradiated neonatal thymuses into the mice resulted in the development of RITL, most of which were derived from the donor thymocytes [6–8]. These findings suggest that BM, rather than the thymus, is a key organ in the pathogenesis of RILT. However, the mechanisms underlying the prevention of RITL with BMT remain unclear.

A recent study demonstrated that using BM cells from some types of transgenic mice, the suppression of RITL through BMT is not the result of the elimination of tumor cells by antitumor immunity, but rather could be the result of the suppression of radiation-induced lymphomagenesis in the thymus by competing with tumor-initiating cells for thymic niches [9].

Based on this study, we measured the number of cells at various stages of differentiation within the thymus of both the donor and recipient using the RITL model transplanted with BM cells obtained from enhanced green fluorescence protein (eGFP) transgenic mice [10] to investigate the cellular interactions within the thymic niches. These BMT experiments allowed us to determine whether the cells migrating to the thymus were of recipient or donor origin.

## MATERIALS AND METHODS

### Animal experiments

All animal experiments were approved by the Institutional Animal Care and Use Committee of Experimental Animals of National Institutes for Quantum Science and Technology.

### Mice

Five-week-old female C57BL/6NCrl mice were obtained from Charles River Laboratories Japan, Inc. (Yokohama, Japan). Five-week-old female C57BL/6-Tg (CAG-EGFP) transgenic (eGFP-tg) mice were purchased from Japan SLC (Hamamatsu, Japan) and maintained in specific pathogen–free conditions. We did not breed the mice. Four C57BL/6NCrl mice were used as the unirradiated control group and five C57BL/6NCrl mice were used in each of the TBI and TBI + BMT groups.

### Total body irradiation

Unanesthetized 6-week-old C57BL/6NCrl mice were X-irradiated with four 1.8 Gy fractions (total dose: 7.2 Gy) at weekly intervals using an X-ray generator (TITAN) operating at 200 kV and 20 mA with a filter consisting of 2.0 mm aluminum and 0.5 mm copper (Shimadzu, Kyoto, Japan) on the same floor of the animal room. The dose rate was ~0.65 Gy/min.

### Bone marrow transplantation

Three hours after the last TBI irradiation, we intravenously injected BM cells (2 × 10^7^ cells/mouse) isolated from two non-irradiated eGFP-tg mice. BM cells were collected as follows. The tibia and femur of both hind legs of each mouse were isolated by sharp dissection. Bones were placed in phosphate-buffered saline (PBS). Thereafter, the ends of the bones were cut at the epiphysis, and the bone marrow was flushed from the bones using PBS and a 26-gauge needle, and then collected in a 15 ml tube. Red blood cells in the bone marrow were lysed using RBC lysis buffer (Thermo Fisher Scientific, San Diego, CA) following the manufacturer’s instructions.

### Cell preparation from thymus tissues

Thymus tissues were dissected from mice and minced. Single-cell suspensions were obtained by digestion with 125 U/ml collagenase Type IV (Worthington Biochemical, Lakewood, NJ) and 60 U/ml DNase I Type IV (Sigma-Aldrich, St. Louis, MO) in PBS for 30 min at 37°C.

### Flow cytometry

Data from the fluorescence intensities of the stained cells were acquired on an SA3800 cell analyzer (Sony, Tokyo, Japan) and analyzed using Kaluza software (Beckman Coulter, Brea, CA). The following antibodies were used: PE-anti-CD4 monoclonal antibody (Mab) (clone GK1.5) was purchased from Thermo Fisher Scientific. The APC-anti-CD8 MAb (clone 53–6.7), Alexa Fluor 700-anti-CD25 MAb (clone PC61), PE-Cy7-anti-CD44 MAb (clone IM7) and purified anti-CD16/32 MAb (Fc block, clone 93) were purchased from BioLegend (San Diego, CA). Cells were blocked with 10 μg/ml purified anti-CD16/32 MAb before staining with the desired combination of fluorescence-labeled MAbs at 10 μg/ml (5 μg/10^6^ cells) in 2% bovine serum albumin and 0.05% azide in PBS for 20 min at 4°C. Cells were washed. Data were collected from 1 × 10^5^ thymocytes using an SA3800 cell analyzer. The gating strategy for the data analysis is shown in Fig. 3.

### Statistical analysis

Data were analyzed for statistical significance using Student’s *t* test or one-way analysis of variance (ANOVA) followed by the Tukey–Kramer test with Microsoft Excel software. Statistical significance was set at 0.05.

## RESULTS AND DISCUSSION

**Fig. 1:**
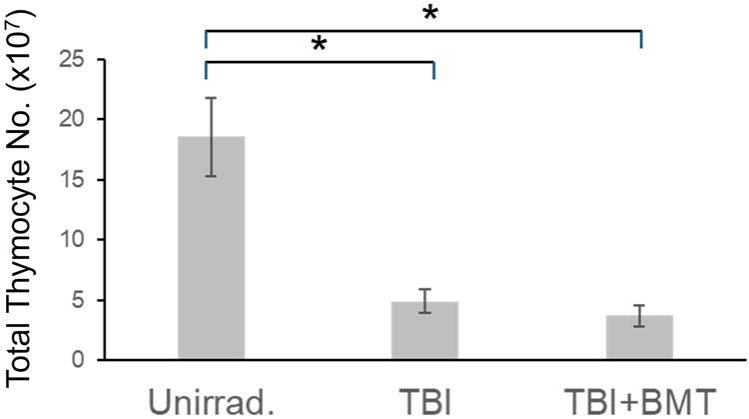
Thymocyte counts 10 days after TBI or TBI + BMT. Data represent mean ± standard deviation (SD) from unirradiated (Unirrad.) controls (*n* = 4), TBI and TBI + BMT mice (each, *n* = 5). ^*^P < 0.05 (one-way ANOVA Tukey–Kramer test).

We measured thymic cell counts in the TBI and TBI + BMT groups 10 days after the last TBI or BMT irradiation. Thymic cell numbers drastically decreased in TBI [4.9 ± 1.0 × 10^7^, mean ± standard deviation (SD)] or TBI + BMT (3.7 ± 0.9 × 10^7^, mean ± SD) mice compared to non-irradiated control (18.6 ± 3.5 × 10^7^, mean ± SD) mice (Fig. 1). BMT hardly increased the number of thymocytes in TBI mice. The thymic cell depletion observed was consistent with a previous study showing thymic atrophy and depletion of cell supply from the BM [11].

**Fig. 2:**
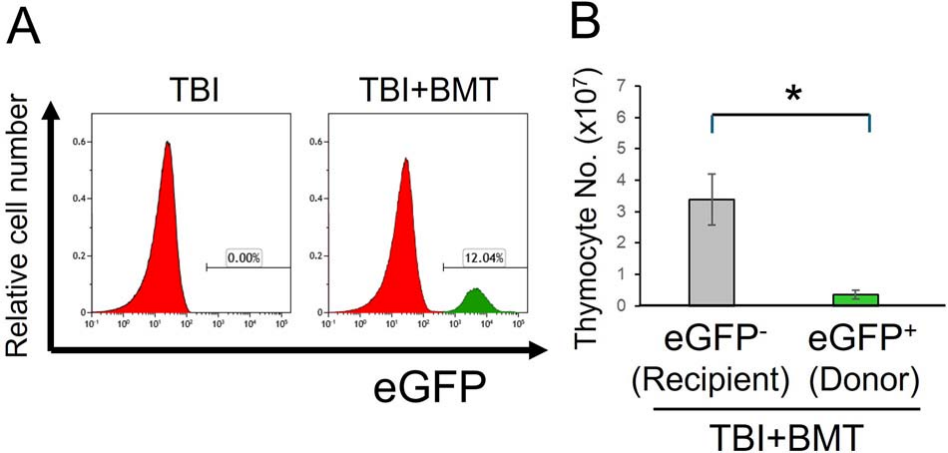
Transplanted bone marrow-derived cell infiltration and thymocyte counts post-TBI and BMT. (**A**) Histograms depicting the frequency of eGFP^+^ (donor-derived) cells in the thymus post-BMT. (**B**) Numbers of eGFP^+^ (donor) and eGFP^−^ (recipient) cells in TBI + BMT mice. Data represent mean ± SD for TBI and TBI + BMT groups (each, *n* = 5). ^*^P < 0.05 (Student’s *t*-test).

Subsequently, to observe the number of transplanted eGFP^+^ BM cells and their differentiated cells in the thymus, we measured the proportion of eGFP^+^ cells in the thymus using flow cytometry and calculated the actual number of each cell type. The results showed that ~10% of eGFP^+^ cells occupied the thymocytes on post-transplantation Day 10 (Fig. 2A). Although eGFP^+^ cells infiltrated the thymus in the TBI + BMT mice, the total number of thymocytes was unchanged between the TBI and TBI + BMT mice (Fig. 1). In TBI + BMT thymuses, eGFP^−^ cells existed significantly more than eGFP^+^ cells (Fig. 2B).

**Fig. 3:**
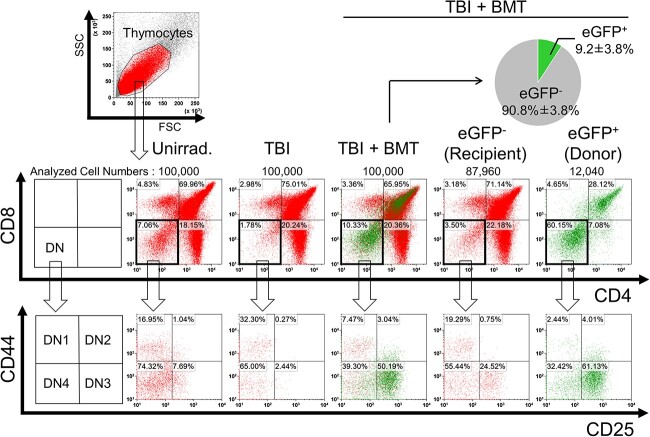
Effects of transplanted BM-derived cells on DN thymocyte subpopulations in mice subjected to TBI. Representative flow cytometry analysis data illustrating the distribution of CD4 and CD8 populations and the four DN subpopulations (DN1, DN2, DN3 and DN4) identified by CD44 and CD25 expression in the thymi of three groups of mice. Analyzed cell numbers are shown. It should be noted that the analyzed cell numbers were different between the eGFP^−^ and eGFP^+^.

**Fig. 4:**
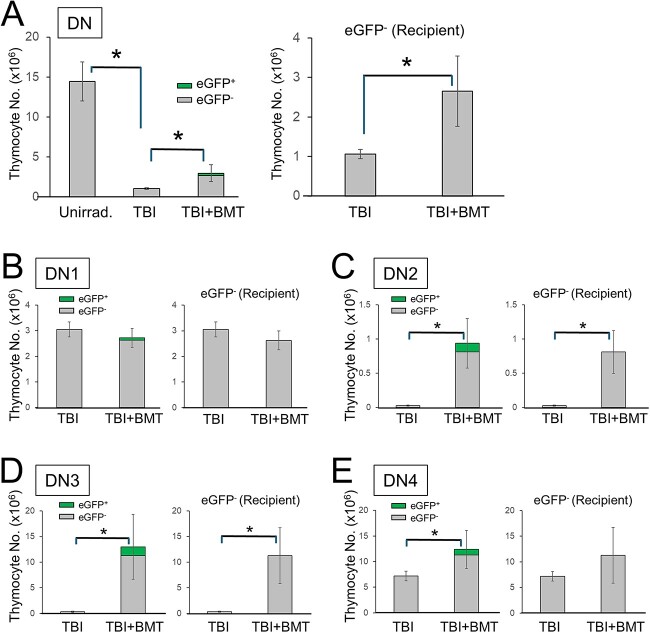
Recipient’s DN population, especially DN2 and DN3, appeared to be significantly recovered in TBI + BMT mice. Absolute counts of (**A**) DN, (**B**) DN1, (**C**) DN2, (**D**) DN3 and (**E**) DN4 subpopulations. Data are presented as mean ± SD from unirradiated controls (*n* = 4) and both TBI and TBI + BMT groups (each, *n* = 5). ^*^P < 0.05 (one-way ANOVA Tukey–Kramer test for three groups or Student’s *t*-test for two groups).

Figures 3 and 4 show the results of the flow cytometric analyses and the absolute thymocyte numbers calculated from these analyses, respectively. Analyses of CD4 and CD8 populations revealed a dramatic decrease in double negative DN (CD4^−^CD8^−^) thymocytes in TBI mice on Day 10 after the last irradiation (Figs 3 and 4A). In particular, the DN2 (CD25^+^CD44^+^) and DN3 (CD25^+^CD44^−^) subpopulations in the thymus of TBI mice almost disappeared, which was different from that of the non-irradiated controls (Fig. 3), although DN1 and DN4 were still present in TBI mice (Fig. 3). This is consistent with the results of a previous study analyzing mice irradiated with daily irradiation of 1.8 Gy × four times [12]. The reason why only the DN2 and DN3 populations, which are the intermediate populations of DN differentiation, were depleted is currently unknown.

This observation is consistent with previously reported differentiation and proliferation rates of thymus-homing precursor cells [13]. Progenitor cells migrating from the bone marrow to the thymus remain at the DN1 stage for 9–10 days, progress to the DN2 stage for 2 days and subsequently to the DN3 stage for 4 days, eventually differentiating into DN4 and DP cells [13]. The lack of differentiation into DN2 and DN3 cells on Day 10 post-TBI may be due to cell death occurring at all stages of cell differentiation; neither the surviving immature cells nor the progenitor cells that migrated from the bone marrow to the thymus afterward differentiated into these stages.

In contrast, DN populations, especially DN2 and DN3, were apparently recovered in TBI + BMT mice (Figs 3 and 4C and D), although the numbers of DN1 populations in the TBI and TBI + BMT groups were unchanged (Fig. 4B). These data suggest that the restoration of the DN population by BMT, especially DN2 and DN3, may be involved in preventing RITL development. Subsequently, the DN subpopulations derived from donor eGFP^+^ BM cells in TBI-BMT mice were analyzed. Surprisingly, the restored DN2 and DN3 cells in TBI-BMT mice were mainly derived from recipient cells and not from eGFP^+^ donor cells (Fig. 4C and D).

Thymus regeneration is believed to occur in a biphasic pattern, where the proliferation of the remaining immature cells is followed by the proliferation of BM cells after radiation exposure [14]. Sunaoshi *et al*. showed that the peaks in DN cell numbers were observed twice, between days 2–5 and 9–11 after irradiation, although the mouse strain or radiation dose was different from that in our study [15]. These data suggest that in the TBI + BMT mice, the transplanted eGFP^+^ BM cells migrate into the thymus and efficiently differentiate and proliferate during the second phase of regeneration.

Currently, it is unclear why the increased DN2 and DN3 populations in the TBI + BMT group were derived from the recipient (eGFP^−^) rather than from the donor (eGFP^+^) (Fig. 4C and D). Thymus-migrated precursor cells proliferate and differentiate via signaling pathways, such as IL-7/IL-7R and Notch1/Deltex4 [16, 17]. We speculate that the few precursor cells that persisted in the post-TBI thymus may have developed into DN2 and DN3 cells, influenced either directly by transplanted BM cells that entered the thymus or by the environmental conditions at their thymic locations, mediated by differentiation signals from transplanted BM cells. Donor BM cells may directly or indirectly interact with recipient intrathymic progenitors, causing them to differentiate into certain cells that can eliminate atypical intrathymic cells, which are probably the precursors of RITL.

Previous studies have demonstrated that radiation-induced lymphoma is significantly suppressed by inhibiting p53-mediated apoptosis [12, 18–21]. This could be because BM-derived hematopoietic stem/progenitor cells are protected from radiation injury. As a result, niche competition in the thymus occurs, and tumor-initiating cells are removed. Transplantation of BM cells from either Rag2^−/−^ mice or Rag2^−/−^; common gamma chain (γc)^−/−^ mice, which have defects in occupying thymic niche beyond DN3 and DN2, respectively, could not prevent RITL [9], suggesting that BM cells suppress radiation-induced lymphomagenesis in the thymus by competing with tumor-initiating cells for thymic niches beyond the DN3 stage.

In summary, our findings strongly suggest that defects in T-cell development are critical for RITL pathogenesis, and that restoration of T-cell development is involved in RITL prevention.
